# Predicting sugar intake using an extended theory of planned behavior in a sample of adolescents: The role of habit and self‐control

**DOI:** 10.1002/brb3.3200

**Published:** 2023-08-03

**Authors:** Daniel J. Phipps, Martin S. Hagger, Kyra Hamilton

**Affiliations:** ^1^ Faculty of Sport and Health Sciences University of Jyväskylä Jyväskylä Finland; ^2^ School of Applied Psychology Griffith University Brisbane Australia; ^3^ Department of Psychological Sciences University of California ‐ Merced Merced USA; ^4^ Health Sciences Research Institute University of California‐, Merced Merced USA; ^5^ Menzies Health Institute Queensland Griffith University Gold Coast Australia

**Keywords:** adolescents, habit, motivation, self‐control, sugar, theory of planned behavior

## Abstract

**Introduction:**

High levels of sugar intake are associated with multiple maladaptive health outcomes in adult and younger populations. Identifying the psychological determinants of sugar intake in adolescents, and the processes involved, may help identify potentially modifiable targets and inform intervention development. We tested the predictions of an extended theoretical model based on the theory of planned behavior (TPB), which specified social cognition constructs, habit, and self‐control as correlates of sugar intake in an adolescent sample.

**Methods:**

Adolescents aged 12 to 14 years (*N* = 88) recruited via a survey panel company and consenting to participate in the study completed online self‐report measures of constructs from the TPB alongside measures of habit and self‐control. One month later, participants completed a follow‐up measure of free‐sugar intake. Hypothesized effects of our proposed extended model were tested using partial least squares structural equation modeling.

**Results:**

We found statistically significant effects of attitude, subjective norm, and perceived behavioral control on sugar intake intentions. We also found significant effects of habit and self‐control on sugar intake measured at follow‐up, but no effect for intention. Perceived behavioral control moderated the intention–behavior relationship such that intention effects on behavior were larger when perceived behavioral control was high. However, self‐control did not moderate the intention–behavior relationship.

**Conclusion:**

Results indicate that sugar intake in this sample was a function of habits and self‐control, and the effect of sugar intake intentions was conditional on perceived behavioral control. Results contribute to an evidence base of determinants and associated processes that relate to sugar intake in adolescents and may signal potentially modifiable targets for intervention.

## Graphical abstract

Acknowledging the deleterious effects of excess sugar intake for the health of adolescents, the present study aimed to identify the psychological determinants of sugar intake in a sample of adolescents using an extended theoretical model. The research adopted a correlational prospective design, and participants completed self‐report measures of key psychological factors and sugar intake. Findings indicated that attitudes, social norms, and perceived control were related to sugar intake intentions, and habit and self‐control, but not intentions, were related to sugar intake. Intentions were related to sugar intake only in those with high perceived control.

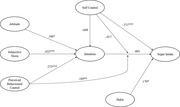

## 1 INTRODUCTION

There are well‐established relations between high levels of “free” sugar consumption in the diet^1^ and deleterious health conditions such as cardiovascular disease, obesity, and poor dental health (Malik et al., 2010), yet population levels of sugar intake remain at levels likely to present a substantive risk to health (Australian Bureau of Statistics, 2016). A growing body of research has aimed to identify the modifiable predictors of excess dietary sugar intake with the goal of creating evidence‐based, theoretically driven behavior change interventions aimed at curbing sugar intake (Hagger et al., 2020). To date, the majority of such research has focused on adult populations. However, there is also evidence that high risk dietary intake patterns develop in childhood and adolescence, and the dietary behaviors adopted early in life may be related to diet in adulthood and obesity in later life (Ludwig et al., 2001; Viner & Cole, 2006). Consequently, it is important to investigate the modifiable predictors of poor dietary behaviors, such as sugar intake, in younger samples in order to inform policy and behavior change strategies which may have health implications for later life.

The key theoretical framework that has been applied to predict health behavior is the theory of planned behavior (TPB; Ajzen, 1991; see also Hagger, 2019). The TPB posits that individuals form intentions to perform a given target health behavior based on their attitude (beliefs about whether the behavior would result in positive or negative outcomes or feelings), subjective norm (beliefs about whether significant others would approve or disapprove of the behavior), and perceived behavioral control (beliefs regarding efficacy or control over the behavior). Intention is hypothesized to be the most proximal predictor of behavior and is hypothesized to have a stronger effect on behavior when individuals perceive the behavior is under their volitional control (Hagger et al., 2022). That is, individuals are more likely to act in accordance with their intentions when they perceive high or complete control over the behavior. To date, studies testing TPB hypotheses have supported its predictions in a variety of health behaviors (Hagger & Hamilton, 2023; Hamilton et al., 2019, 2020, 2021; McEachan et al., 2011), including dietary behaviors in general (Brown et al., 2021) and sugar intake in particular (Hagger et al., 2017; Phipps et al., 2020).

There has also been support for the predictions of the TPB in health behavior and in child and adolescent samples (Hamilton et al., 2020; McEachan et al., 2011), including in predicting young people's dietary behaviors (Riebl et al., 2015). It is important to consider that the relative effects of the TPB constructs on intention and behavior may differ in younger samples compared with adults. For example, a meta‐analysis found that the size of the effect of subjective norm on dietary intentions was small in adult samples and much larger in adolescent samples (McEachan et al., 2011). This suggests younger samples may be more subject to the influence of social pressures, particularly those from friends and peers (Gibbons et al., 2009), and may rely less on their perceptions of long‐term health benefits when it comes to making decisions to engage in health behaviors. These differences imply that research applying the TPB to predict dietary choices in adult samples may not be directly translatable to children and adolescents, particularly in respect to the relative contribution of the different belief‐based constructs to intention formation. Consequently, the investigation of the belief‐based correlates of intentions and behavior in younger samples represents a potentially important avenue for informing age‐specific behavior change strategies.

It is also important to note that the TPB may not encompass all the potential determinants and associated processes involved in explaining variance in health behaviors (Hagger et al., 2017; Phipps et al., 2021, 2022). For example, the TPB is often criticized for not outlining all potential conditions that account for the relationship between intentions and behavior, particularly the reported intention–behavior “gap.” The latter has been indicated by research demonstrating that the effect of intention on behavior is often modest in size (Sniehotta et al., 2014). One potential explanation for the intention–behavior “gap” may be that individuals have insufficient self‐regulatory capacity to act on their intentions (Conner & Armitage, 1998). For example, an individual who intends to minimize their sugar intake in the coming week may be more likely to act on that intention if they have higher levels of self‐control. That is, a capacity to inhibit impulse‐driven responses that might drive spontaneous, well‐learned, reward‐driven sugar intake. Individuals with high self‐control, therefore, may have high capacity to resist tempting situations which may compel them to overconsume sugary foods, or, at least, provide them with the impetus to develop goal‐directed strategies so as not to get into tempting situations in the first place (Pfeffer & Strobach, 2022).

In addition, the TPB may not be fit‐for‐purpose as an account of frequently performed, day‐to‐day behaviors, such as dietary choices, as evidenced by relatively modest proportion of explained variance identified in prior research (e.g., Hagger & Hamilton, 2023; Hamilton et al., 2020; McEachan et al., 2011). That is, behaviors with which the individual has considerable past experience and that they may have performed repeatedly under the same conditions and in the presence of the same cues or contextual factors. This is likely because the TPB is adequate in accounting for behaviors that tend to necessitate deliberative, reasoned consideration of the merits and detriments of performing the behavior in future. However, such cognitively demanding deliberation is likely only necessary in situations where an individual has relatively little prior experience with the behavior, or in specific instances that are out of the ordinary, or where the behavioral decision is complex or dependent on a large a number of contingencies (Bargh & Chartrand, 1999; Verplanken & Orbell, 2003). By contrast, the theory may be less effective in accounting for behavior in cases where an individual has considerable prior experience of performing it and in stable contexts or in the presence of commonly occurring cues or contingencies. Such behaviors are more likely to be enacted with relatively little deliberation and are, instead, more likely to be instigated or performed with much less cognitive deliberation. As such, variance in these behaviors is more likely to be explained by constructs that represent efficient, routinized, context‐, or cue‐dependent processes such as habit.

In the context of children and adolescents, it is feasible that health behavior like sugar intake may be developed to be habitual due to frequent, context stable occurrences. Thus, sugar consumption in children may be more likely to be experience as habitual rather than as a result of the reasoned consideration of the merits of a behavior, the social influences to perform it, and their capacity to do so. There is evidence to support this hypothesis. Habit as a construct has been consistently associated with adolescent health behavior (de Bruijn & van den Putte, 2009; Kremers & Brug, 2008; Kremers et al., 2007), and, for some behaviors (e.g., soft drink intake, TV viewing; Kremers & Brug, 2008), habit has tended to have larger effects on behavior than intention. However, investigations into the effects of habit on health behavior in younger samples lacks the consistent evidence present in research on adults, particularly when considering effects of habit alongside constructs that represent reasoned processes like those featured in the TPB (e.g., Hagger et al., 2023).

The dearth of research in children and adolescents on the relative effects of constructs that represent reasoned processes, such as those offered in the TPB, conditions that determine the intention–behavior relationship, and constructs that represent nonconscious, habitual processes constitute the impetus for the current study. Specifically, we aimed to investigate the predictors of free‐sugar intake in a sample of adolescents using an extended TPB, which incorporated effects of self‐control and habit. We hypothesized effects of attitude, subjective norm, and perceived behavioral control on sugar intake intentions, and an effect of intention on prospectively measured sugar intake, consistent with the TPB. Further, we predicted that perceived behavioral control would moderate the intention–behavior relationship, such that the relationship would be stronger in those reporting higher levels of perceived behavioral control. In addition, we also predicted that self‐control and habit would both predict sugar intake directly, and that self‐control would also moderate the intention–behavior relationship, such that the relationship would be stronger among those endorsing high levels of self‐control.

## 2 MATERIALS AND METHODS

### 2.1 Participants

An initial sample of 183 adolescents aged 12 to 14 years from Australia was recruited from the general population using a panel company. We targeted this age group because the transition to adolescence likely marks a key period in which young people gain increased independence from home life and parental supervision, and begin to establish behavioral patterns for sugar consumption that are less regulated by their parents and more influenced by context, preference, and peers. As a consequence, young people in this age group may begin to form strong beliefs that become reinforced and develop into habits, which once established may be linked to potential adverse long‐term health outcomes. All materials were administered via the Qualtrics online survey software, taking approximately 15 min to complete. After both parent and child provided informed consent, participants were presented with a basic definition of “free” dietary sugar, and then completed measures of habit, self‐control, and the TBP. One month later, participants were recontacted via the panel company to complete a survey of their sugar intake over the previous month. Ninety five participants did not return to complete the final survey, resulting in a final sample of 88 adolescents (*M*_Age_ = 13.65, SD_Age_= 0.61, 41 male, 45 female, two preferred not to say). Participants in the final sample did not differ from those who did not complete the follow‐up measure of behavior in terms of their age (*t*(181) = 1.60, *p* = .112, *d* = .24), gender (*χ* ^2^(3) = 5.86, *p* = .119), or baseline study variable scores (*Λ* = .989, *F*(6, 176) = 0.32, *p* = .925, *η*_p_^2^ = .01). All procedures were approved by Griffith University Human Research Ethics Committee.

### 2.2 Measures

Participants’ sugar intake habit was assessed using the self‐report behavioral automaticity index (SRBAI; Gardner et al., 2012; Verplanken & Orbell, 2003). The SRBAI requires participants to respond to four items (e.g., “Consuming foods and drinks high in free sugar as part of my daily diet is something I do without thinking”) with responses provided on 5‐point scales (1 = *strongly disagree* to 5 = *strongly agree*).

Participants’ level of trait self‐control was assessed using the IPIP‐HEXACO self‐discipline scale (Ashton et al., 2007). The scale comprised of 10 items (e.g., “I have difficulty starting tasks”), scored on 5‐point scales (1 = *strongly disagree* to 5 = *strongly agree*).

All TPB belief‐based items used to tap attitude, subjective norm, and perceived behavioral control constructs were developed according to published guidelines for assessing each construct (Ajzen, 2002). Measures following these guidelines have previously been used successfully to tap beliefs in adolescent samples (e.g., Baker & White, 2010).

Attitude was assessed using five items each scored on 5‐point semantic differential scales (Ajzen, 2002) (e.g., “For me, consuming foods and drinks high in free sugar as part of my daily diet in the next month would be… *bad*‐*good*”).

Subjective norm was assessed using three items (Ajzen, 2002) (e.g., “Most people who are important to me would approve of me consuming foods and drinks high in free sugar as part of my daily diet”). Each item was scored on a 5‐point scale (1 = *strongly disagree* to 5 = *strongly agree*).

Perceived behavioral control was assessed using four items (Ajzen, 2002) (e.g., “If I wanted to, I could easily consume foods and drinks high in free sugar as part of my daily diet”), each scored on 5‐point scales (1 = *strongly disagree* to 5 = *strongly agree*).

Intention was assessed using three items (Ajzen, 2002) (e.g., “It is likely that I will consume foods and drinks high in free sugar as part of my daily diet in the next month”). Each item was scored on a 5‐point scale (1 = *strongly disagree* to 5 = *strongly agree*).

Sugar intake at the follow‐up time point was measured using the sugar section of the Dietary Fat and Free Sugar food frequency questionnaire (DFS; Francis & Stevenson, 2013). The DFS requires participants to respond to how often they consumed 12 high sugar food exemplars (e.g., “Chocolate”) on 5‐point scales (1 = *less than once a month* to 5 = *5+ times a week*).

Complete study measures are available in Appendix A (Supplemental Material).

### 2.3 Data analysis

Hypothesized effects of the proposed model were analyzed using linear partial least squares structural equation modeling with the WarpPLS analytic software (Kock, 2018), with standard errors calculated via the “Stable 3” method. Calculation of standard errors using the “Stable 3” method in PLS‐SEM produces results largely consistent with a bootstrapping approach from standard ordinary least squares regression and path analysis (for a detailed explanation, see Kock, 2014). The analysis yields a number of salient global model fit and quality indices including Tenenhaus’ GoF index (GoF), Simpson's paradox ratio (SPR), the R‐squared contribution ratio (RSCR), and the average block variance inflation factor (AVIF). Values exceeding 0.36 for the GoF indicate large effect sizes, associated with good model quality, and values exceeding 0.70 and 0.90 for the SPR and RSCR, and less than 3.3 for the AVCIF, also indicate good fit and model quality. Effects in the model were expressed as standardized parameter estimates with 95% confidence intervals.

## 3 RESULTS

**FIGURE 1:**
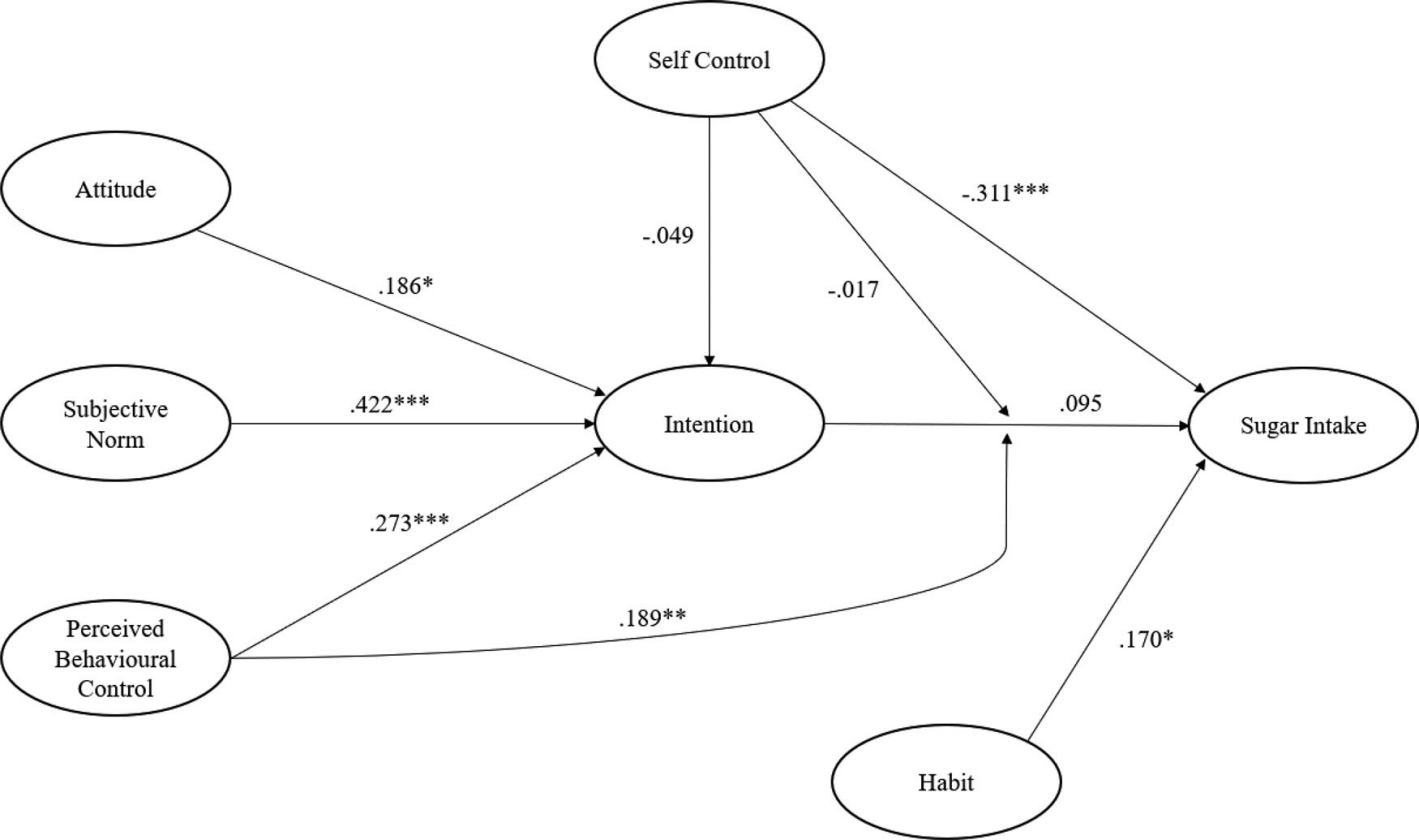
Diagram summarizing proposed effects among the model predicting sugar intake. Effects expressed as standardized parameter estimates. *p < .05 **p < .01 ***p < .001.

Zero‐order correlations, Cronbach's alpha reliability statistics, and descriptive statistics are presented in Table 1. Our proposed model predicting sugar intake exhibited good fit with the data and good model quality (GoF = .430, SPR = .889, RSCR = 1.00, AVIF = 1.40; see Figure 1), and predicted 36.6% of the variance in intention and 21.5% of the variance in sugar intake. All indicator items loaded significantly onto their respective constructs (*p*s < .032), with the exception of a single DFS item (item 10: “milkshakes, hot chocolates, etc.”, *p* = .172), which missed the conventionally accepted threshold for statistical significance but was retained regardless for consistency with the prior published measure. Model standardized parameter estimates are presented in Table 2. We found statistically significant direct, positive effects of attitude, subjective norm, and perceived behavioral control on intention to consume sugar, but no significant effect for self‐control. However, we found significant negative effects of self‐control and positive effects of habit, but not intention, on prospectively measured sugar intake. Importantly, the effect of intention on sugar intake was positively moderated by perceived behavioral control. This interaction effect indicated that the intention–behavior relationship was stronger among participants endorsing higher levels of perceived behavioral control. The interaction effect is illustrated in the figure in Appendix B (Supplemental Materials). By contrast, self‐control did not moderate the intention–behavior relationship.

**TABLE 1:** Latent variable correlations, internal consistency statistics, and descriptive statistics for study variables.

Variable	1	2	3	4	5	6	7	*M*	SD	*α*
1. Self‐control	–							2.63	0.55	.511
2. Habit	−.188	–						2.82	1.02	.895
3. Attitude	−.084	.312^b^	–					2.87	0.85	.690
4. PBC	−.082	.159	.120	–				3.72	0.78	.866
5. Subjective norm	.077	.196	.187	.150	–			2.86	0.93	.835
6. Intention	−.054	.529^c^	.302^b^	.363^c^	.494^c^	–		3.09	0.96	.897
7. Sugar Intake	−.343^b^	.302^b^	.247	−.050	.183	.201	–	2.13	0.54	.735

PBC, perceived behavioral control.

^a^ *p* < .05.

^b^ *p* < .01.

^c^ *p* < .001.

**TABLE 2:** Standardized parameter estimates for proposed model effects.

Effect	*β*	*p*
Attitude **→** intention	.170	.018
Subjective norm **→** intention	.422	<.001
Perceived behavioral control **→** intention	.273	<.001
Self‐control **→** intention	−.049	.270
Self‐control → sugar Intake	−.311	<.001
Intention **→** sugar Intake	.095	.118
Self‐control × intention → sugar Intake	−.017	.415
Perceived behavioral control × intention → sugar intake	.189	.010
Habit → sugar Intake	.170	.015

## 4 DISCUSSION

The current study aimed to test an extended TBP in the context of sugar intake in a sample of adolescents. Findings revealed effects of attitude, subjective norm, and perceived behavioral control on sugar intake intention, consistent with TPB predictions, but no effect of intention on behavior. However, we found direct effects of habit and self‐control on behavior, as predicted. Perceived behavioral control moderated the intention–behavior relationship, as predicted in the TPB, but, contrary to our hypothesis, self‐control did not.

Although attitude, subjective norm, and perceived behavioral control were related to adolescents’ sugar intake intentions as predicted in the TBP (Ajzen, 1991), intentions were not related to sugar intake. While this is in contrast to previous theory and research (e.g., Riebl et al., 2015), it is important to note that the effect of intention on behavior was moderated by perceived behavioral control, such that intention was associated with behavior only among those reporting high levels of perceived behavioral control. This moderating effect of perceived behavioral control on the intention–behavior relationship is a core prediction specified by Ajzen, but is not often tested, despite support for this effect in the literature (Hagger et al., 2023). Adolescents, therefore, were more likely to enact their intentions to reduce their sugar intake when they viewed doing so as achievable and when they perceived few barriers or had sufficient contingency plans to overcome potential barriers to doing so. By contrast, adolescents were less likely to reduce their sugar in take when they perceived the behavior as less achievable, and when they perceived salient barriers to doing so or they that had less capacity to overcome the barriers.

In terms of the current behavioral context and study population, the latter explanation is of particular relevance, as parents, caregivers, and schools likely all have a degree of control over adolescents’ dietary choices. For example, parents’ or caregivers’ rules regarding sugar intake, attempts to limit the availability of sugar, and their own sugar intake behaviors have all been associated with reduced sugar intake in adolescents (Van de Gaar et al., 2017; Yee et al., 2019). Further, parental control has been found to predict adolescents dietary choices beyond the effects of the TPB (Karimi‐Shahanjarini et al., 2012). Thus, our finding that intention did not predict sugar intake in adolescents reporting low levels of perceived behavioral control may be attributable to their parents’ control beliefs and attempts to restrict intake levels that inhibit the child's ability to effectively act on their stated intentions. However, adolescents’ beliefs regarding parental control, and parents’ actual restriction of their child's sugar intake, were not measured directly in the current study. Future research is required to examine whether perceived or actual parental control is the prevailing underlying factor that moderates the relationship between adolescents’ intentions and their sugar intake.

High self‐control was also associated with a lower level of sugar intake. Adolescents with higher levels of self‐control are likely to have better capacity to suppress impulse‐driven responding and may have a better inherent capacity to inhibit their intake of high sugar foods (Hagger, Hankonen et al., 2019), resulting in a direct effect of self‐control on behavior not mediated by beliefs (Hagger, Gucciardi et al., 2019; Shahzalal & Adnan, 2022). However, self‐control did not moderate the intention–behavior relationship. Based on theories of self‐control (Carver & Scheier, 1982), we expected that those high in self‐control would be more likely to resist temptations and develop appropriate regulatory strategies to manage situations where they might eat or overeat sugary foods, and, therefore, less likely act on intentions to avoid consuming sugar. However, studies testing this moderation effect have produced mixed evidence (Pfeffer & Strobach, 2017, 2022; Schöndube et al., 2017; Van Gelderen et al., 2015). In light of contrasting findings, it is possible that the presence of an interaction between self‐control and intention may be conditional on other factors which enable individuals to develop self‐regulatory resources, such as stress, cognitive load, or sensitivity to behavior related cues (Muraven et al., 2002; Pfeffer & Strobach, 2017; Pfeffer et al., 2020).

Last, we observed an effect of habit on sugar intake, consistent with our expectations and previous research in adult samples (Gardner et al., 2011; Hagger et al., 2017, 2023; Phipps, Hagger et al., 2021). Dietary choices are likely to occur frequently and in stable contexts (e.g., eating a sweet snack after school, drinking a soft‐drink while watching a movie), thus promoting the development of habit (Danner et al., 2008; Gardner & Lally, 2018). Once a habit is developed, behavior is likely enacted automatically as a result of learned cue‐behavior scripts (Gardner, 2012), minimizing the need for excessive cognition. These findings add to the body of evidence supporting the importance of habit on adolescent behavior (de Bruijn & van den Putte, 2009; Kremers & Brug, 2008; Kremers et al., 2007) and may be of particular note in the domain of public health, as habits are generally considered to be deep‐seated, stable associations (Gardner et al., 2021; Lally & Gardner, 2013). As such, it is possible habit may, in part, account for the progression of poor health behaviors from childhood and adolescents into adulthood often noted in public health statistics (Ludwig et al., 2001; Viner & Cole, 2006). Thus, while additional research is needed to investigate the longitudinal effects of habit formation in early life, the current findings indicate the potential value of developing and promoting healthy habits from an early age.

While the current study included several novel features, it is not without limitations. Although the current study employed a validated measure of behavior (Francis & Stevenson, 2013), self‐reported measures are at risk of being affected by response bias or poor recall (Subar et al., 2015). Future research may seek to replicate the tested model using observational measures of sugar intake to corroborate current findings. Further, sample attrition across data collection occasions was higher than expected. However, comparisons between the final sample and those who did not complete the follow‐up survey indicated trivial differences, and we had sufficient statistical power to detect modest effect sizes that we deemed of consequence in our outcome variables. Regardless, we advocate that future research seek to replicate the current findings in larger samples with higher statistical power. In addition, caution should be exercised when generalizing these findings broadly. For example, should be noted that the sample is not representative of the population and patterns of sugar intake beliefs may vary between cultures and locations. Thus, it may be prudent to replicate the current research in additional contexts and population groups. Finally, although the internal consistency of the self‐control measure fell below conventionally accepted cutoff values, items from this scale, as well as others, were used to indicate latent constructs that are ostensibly “error free.” Nevertheless, as suboptimal reliability may be associated with additional error, our findings relating to the effects of this construct should be interpreted with this caveat in mind.

## 5 CONCLUSION

The current study aimed to predict sugar intake in adolescents using an extended TPB that incorporated habit and self‐control. Attitude, subjective norm, and perceived behavioral control were all related to intention as predicted. Unexpectedly, intention was not related to behavior. However, we found that perceived behavioral control moderated the effect of intention on behavior. In addition, habit and self‐control were associated with sugar intake, but self‐control did not moderate the intention–behavior relationship. Our findings advance theory by demonstrating key roles for additional constructs that represent nonconscious processes, and explain key effects in the TPB, that is, the intention–behavior relationship. Although the correlational design precludes definitive recommendation for practice, these findings may serve as a starting point for an evidence base of constructs that may serve as candidate targets for behavior change interventions, such as the promotion of habits and support for self‐control.

## CONFLICT OF INTEREST STATEMENT

The authors have no conflict of interest to declare.

### PEER REVIEW

The peer review history for this article is available at https://publons.com/publon/10.1002/brb3.3200.

## Supporting information

Supplemental Materials: Appendix A
Supplemental Materials: Appendix B

## Data Availability

The data that support the findings of this study are openly available in Open Science Framework at https://osf.io/3vrx9/.

## Note

1 In this case, “free” sugar consumption refers to dietary sugar that is added to food products, that is, not inherent or naturally occurring sugar, often referred to as “added sugar” and is present mainly in highly processed foods.
