# Supplementary material for: Predicting sugar intake using an extended theory of planned behavior in a sample of adolescents: The role of habit and self‐control
Source: Brain Behav. 2023 Aug 3;13(10):e3200. doi: 10.1002/brb3.3200 (PMC10570494; doi:10.1002/brb3.3200)

**Supplemental Materials: Appendix A**

*Self-Reported Measures*

| Scale | Items | Responses |
| --- | --- | --- |
| Self-Control | I have difficulty starting tasks  I get my chores done right away  I find it difficult to get down to work  I am always prepared  I often waste my time  I start tasks right away  I tend to postpone decisions  I like to get to work at once  I need a push to get started  I tend to carry out my plans | [1] Strongly Agree to [5] Strongly Disagree  [1] Strongly Disagree to [5] Strongly Agree  [1] Strongly Agree to [5] Strongly Disagree  [1] Strongly Disagree to [5] Strongly Agree  [1] Strongly Agree to [5] Strongly Disagree  [1] Strongly Disagree to [5] Strongly Agree  [1] Strongly Agree to [5] Strongly Disagree  [1] Strongly Disagree to [5] Strongly Agree  [1] Strongly Agree to [5] Strongly Disagree  [1] Strongly Disagree to [5] Strongly Agree |
| Habit | Consuming foods and drinks high in free sugar as part of my daily diet is something I do automatically  Consuming foods and drinks high in free sugar as part of my daily diet is something I do without having to consciously remember  Consuming foods and drinks high in free sugar as part of my daily diet is something I do without thinking  Consuming foods and drinks high in free sugar as part of my daily diet is something I start to do before I realise I’m doing it | [1] Strongly Disagree to [5] Strongly Agree  [1] Strongly Disagree to [5] Strongly Agree  [1] Strongly Disagree to [5] Strongly Agree  [1] Strongly Disagree to [5] Strongly Agree |
| Attitude | For me, to consuming foods and drinks high in free sugar as part of my daily diet in the next month would be… | [1] Bad to [5] Good  [1] Disgusting to [5] Delicious  [1] Nasty to [5] Enjoyable  [1] Unhealthy to [5] Healthy  [1] Worthless to [5] Valuable |
| Subjective Norm | Most people who are important to me would approve of me consuming foods and drinks high in free sugar as part of my daily diet  Most people whose opinions I value think that I should consume foods and drinks high in free sugar as part of my daily diet  Most people who are important to me are consuming foods and drinks high in free sugar as part of their daily diet | [1] Strongly Disagree to [5] Strongly Agree  [1] Strongly Disagree to [5] Strongly Agree  [1] Strongly Disagree to [5] Strongly Agree |
| Perceived Behavioural Control | It is mostly up to me whether I consume foods and drinks high in free sugar as part of my daily diet.  It would be possible for me to consume foods and drinks high in free sugar as part of my daily diet.  I have complete control over whether I consume foods and drinks high in free sugar as part of my daily diet.  If I wanted to, I could easily consume foods and drinks high in free sugar as part of my daily diet. | [1] Strongly Disagree to [5] Strongly Agree  [1] Strongly Disagree to [5] Strongly Agree  [1] Strongly Disagree to [5] Strongly Agree  [1] Strongly Disagree to [5] Strongly Agree |
| Intention | I intend to consume foods and drinks high in free sugar as part of my daily diet in the next month  I expect I will consume foods and drinks high in free sugar as part of my daily diet in the next month  It is likely that I will consume foods and drinks high in free sugar as part of my daily diet in the next month. | [1] Strongly Disagree to [5] Strongly Agree  [1] Strongly Disagree to [5] Strongly Agree  [1] Strongly Disagree to [5] Strongly Agree |
| DFS Food Frequency Questionnaire | Think about the food you’ve eaten over the past month. Remember breakfast, lunch, dinner and eating out. Please select the option that best describes how often you have consumed each of the following food or drink items.  Doughnuts, pastries, or croissants  Cakes or cookies  Ice cream  Chocolate  Spreads including peanut butter, jam, or honey  Pancakes or French toast  Sports drinks (e.g. Gatorade) or energy drinks (e.g. Red Bull)  Soft drink (not including diet)  Milk (full fat only). Include milk in cappuccinos, milkshakes, hot chocolates etc.  Other sweetened beverages (e.g. juice with added sugar, cordial, sweetened teas)  White bread (white bread only) | [1] Less than once a month to [5] 5+ times a week  [1] Less than once a month to [5] 5+ times a week  [1] Less than once a month to [5] 5+ times a week  [1] Less than once a month to [5] 5+ times a week  [1] Less than once a month to [5] 5+ times a week  [1] Less than once a month to [5] 5+ times a week  [1] Less than once a month to [5] 5+ times a week  [1] Less than once a month to [5] 5+ times a week  [1] Less than once a month to [5] 5+ times a week  [1] Less than once a month to [5] 5+ times a week  [1] Less than once a month to [5] 5+ times a week |

**Supplemental Materials: Appendix B**

Moderation effects were further investigated using the inbuilt WarpPLS simple slopes function using a median split of the moderating variable, perceived behavioral control (PBC).


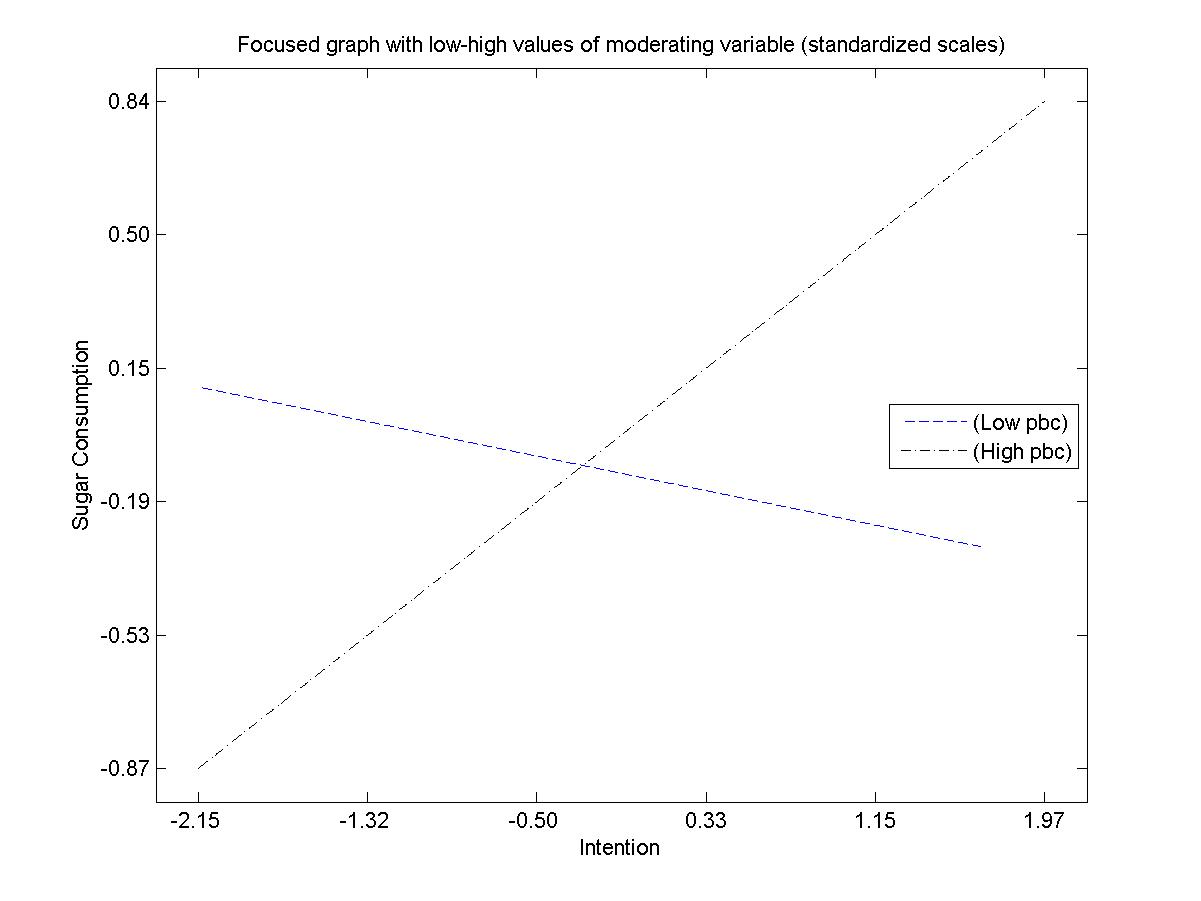

Supplement: Supplementary file 1 — Supplemental Materials: Appendix A Supplemental Materials: Appendix B [file BRB3-13-e3200-s001.docx]
